# A Novel Triple Crosslinking Strategy on Carbon Nanofiber Membranes as Flexible Electrodes for Lithium-Ion Batteries

**DOI:** 10.3390/polym14173528

**Published:** 2022-08-28

**Authors:** Hang Xu, Xinran Hou, Man Gong, Changshu Yang, Jinpeng Luo, Yuluo Chen, Lei Ma, Lang Zhou, Chuanqiang Yin, Xiaomin Li

**Affiliations:** 1 Institute of Photovoltaics, Nanchang University, Nanchang 330031, China; 2 Guangxi Key Laboratory of Information Materials, Guilin University of Electronic Technology, Guilin 541004, China

**Keywords:** electrospinning, crosslinking, carbon nanofiber membranes, flexible electrodes, lithium-ion batteries

## Abstract

In order to solve the problem of low electrical conductivity of carbon nanofiber membranes, a novel triple crosslinking strategy, including pre-rolling, solvent and chemical imidization crosslinking, was proposed to prepare carbon nanofiber membranes with a chemical crosslinking structure (CNMs-CC) derived from electrospinning polyimide nanofiber membranes. The physical-chemical characteristics of CNMs-CC as freestanding anodes for lithium-ion batteries were investigated in detail, along with carbon nanofiber membranes without a crosslinking structure (CNMs) and carbon nanofiber membranes with a physical crosslinking structure (CNMs-PC) as references. Further investigation demonstrates that CNMs-CC exhibits excellent rate performance and long cycle stability, compared with CNMs and CNMs-PC. At 50 mA g^−1^, CNMs-CC delivers a reversible specific capacity of 495 mAh g^−^^1^. In particular, the specific capacity of CNMs-CC is still as high as 290.87 mAh g^−1^ and maintains 201.38 mAh g^−1^ after 1000 cycles at a high current density of 1 A g^−1^. The excellent electrochemical performance of the CNMs-CC is attributed to the unique crosslinking structure derived from the novel triple crosslinking strategy, which imparts fast electron transfer and ion diffusion kinetics, as well as a stable structure that withstands repeated impacts of ions during charging and discharging process. Therefore, CNMs-CC shows great potential to be the freestanding electrodes applied in the field of flexible lithium-ion batteries and supercapacitors owing to the optimized structure strategy and improved properties.

## 1. Introduction

Energy storage technologies have embraced a golden period of development in recent decades as energy demand increases [1,2]. Carbon fiber, an interesting candidate among carbon-based anode materials, has attracted great popularity owing to a series of characteristics such as a high elastic modulus, excellent electrical conductivity, chemical stability and rich sources, which has been extensively researched as an electrode material for flexible lithium-ion batteries (LIBs) [3], lithium-metal batteries [4], lithium-sulfur batteries [5] and supercapacitors [6]. Flexible electrodes are the core component of flexible energy storage devices [7]. Metals with excellent electrical and mechanical properties have been widely employed as current collectors for various electrodes. Generally, active powder materials, conductive additives, and binders have to be coated on the rigid metal foil in the traditional electrode preparation technology. However, the shortcomings of poor mechanical strength and easy detachment from the matrix will result in irreversible deformation during the bending process, which cannot meet the flexibility requirements [8,9]. Moreover, the volume energy density of the electrode is always reduced because of carbon black used as conductive additives and some polymers used as binders in the practical applications [10,11]. Therefore, the development of freestanding flexible electrodes without a binder and a current collector has been a promising strategy for flexible LIBs [12].

Electrospinning is a versatile, simple, and inexpensive fiber manufacturing process that has a very broad potential for energy storage [13,14,15]. The principle is to prepare non-woven products with fiber diameters ranging from the submicron to nanometer scale by using high-voltage electrostatic field forces as a traction force. Electrospinning nanofibers possess many excellent properties, such as porosity, flexibility, high fiber homogeneity, high specific surface area, mild preparation conditions, wide applicability, simple process, and excellent 3D pore structure [16]. Recently, with the maturity of electrospinning technology and abundance of spinnable raw materials, its development in the preparation of flexible anode has greatly expanded the choice of new materials for the anodes of LIBs. Carbon nanofiber membranes prepared by electrospinning exhibit excellent thermal stability, chemical stability, good electrical conductivity, mechanical properties and electrochemical properties, which can shorten diffusion paths of lithium ions and expand the specific surface area of electrode materials [17,18,19]. Electrospinning porous carbon nanofibers with a self-supporting structure do not require a current collector, a conductive agent and a binder when they are directly used as anodes, which can improve the energy density of anodes and simplify preparation process to save cost of production. Compared with slurry coating technology, flexible self-supporting carbon fibers electrodes have bright prospect and enormous challenges [20].

There are many precursors for the preparation of carbon fibers by electrospinning, mainly polyacrylonitrile (PAN) [21,22,23] and polyimide (PI) [24]. PI, a general term for a class of polymers containing imide rings in their main chains, is among the most comprehensive polymeric materials available [25]. Compared with PAN, PI has a higher carbon yield, better mechanical properties and electrochemical properties that have attracted the interest of a wide range of researchers [26]. Hence, CNM derived from PI becomes a new flexible anode for LIBs with great potential due to the benefits of high specific surface area of nanofiber membranes and thermal stability and chemical stability of PI [27].

To improve the electrochemical properties of carbon membranes, various strategies have been tried by many researchers, including N-doping strategies (ammonia [28], melamine [29], and urea [30]) and increasing the specific surface area of samples. Zhao et al. [31] prepared flexible heteroatoms-doped carbon nanofiber membranes with excellent rate and cycle performances, which showed a specific capacity of 695 mAh g^−1^ at 100 mA g^−1^ and a retain value of 245 mAh g^−1^ at 1.5 A g^−1^ after 300 cycles. Jiang et al. [32] reported an electrospun composite nanofiber obtained from polyacrylonitrile/zinc chloride as a precursor. The introduction of ZnCl_2_ can significantly enhance the specific surface area.

However, the preparation of crosslinking carbon nanofiber membranes and their effects on the comprehensive performance are still rarely studied. In particular, the electrochemical properties of anode are strongly correlated with their microscopic morphology, surface properties [33]. Typically, electrospun fibers are stacked to form a binder-free membrane. However, carbon fibers with high porosity usually exhibit physical contact, resulting in blocked electron transfer perpendicular to membranes. Hence, establishing a continuous electron transfer path can enhance the electronic conductivity of carbon membranes and thus improve energy storage properties.

**Figure 1:**
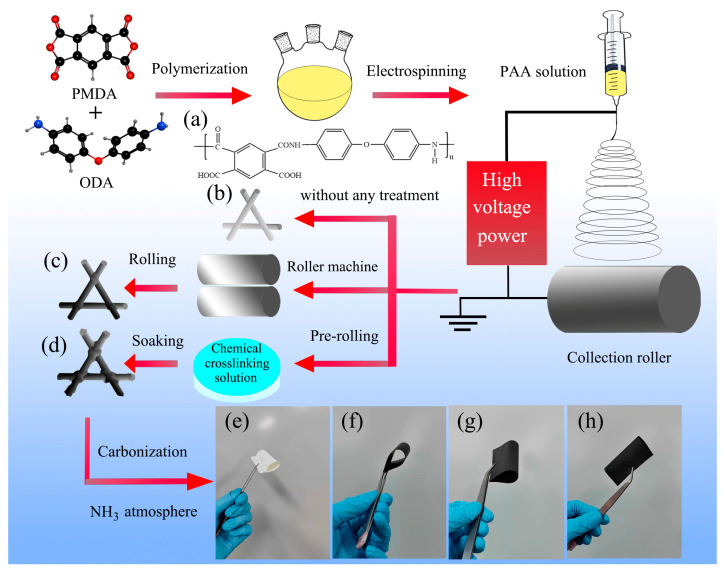
Schematic illustration for fabrication process of carbon nanofiber membranes. (**a**) The chemical structure of PAA (polyimide precursors), (**b**) PAA nanofiber membranes (PAANMs) without any treatment, (**c**) PAANMs-PC, (**d**) PAANMs-CC, corresponding optical photographs of (**e**) PAANMs, (**f**) CNMs, (**g**) CNMs-PC, and (**h**) CNMs-CC in the bent state.

In this work, a simple and novel triple crosslinking strategy of electrospinning CNMs-CC derived from PI was proposed to improve the electrical conductivity of samples used as flexible anode material for LIBs to a certain extent. Firstly, the physical contact of the fibers is made tight by pre-rolling, and then a strong chemical crosslinking structure is formed by the combined action of solvents and imidization reagents as shown in Figure 1. By the way, the effects of a crosslinking structure on the properties of samples were investigated by the characterization analysis of SEM, TG, DSC, XRD, etc. The results show that the introduction of the crosslinking structure effectively improves the loose lap problem and increases the interaction force of fibers. Therefore, the preparation of self-supporting CNMs electrodes with triple crosslinking structures may be confirmed to be a simple and practically applicable strategy for flexible LIBs.

## 2. Experimental

### 2.1. Preparation of Carbon Membranes

A two-step synthesis of polyimide was performed by condensation reaction of the pyromellitic dianhydride (PMDA, ≥99.5%) and the 4,4′-diaminodiphenyl ether (ODA, ≥99.5%) in a molar ratio of 1:1 to synthesize the precursor polyamide acid (PAA) solution. PMDA and ODA were supplied by Tianjin Haopu Chemical Co. Firstly, ODA was dissolved in 100 mL dimethylacetamide (DMAc) and stirred well under nitrogen. DMAc was purchased from Sinopharm Group Chemical Reagent Co. Next, PMDA was added to the solution in several portions for the polymerization reaction and mechanically stirred for 6 h. The total amount of solvent used was 135 g to form a ~10 wt% solution.

Then, the as-obtained PAA was placed in a 5 mL syringe as the spinning solution, and the PAA nanofiber membrane (PAANMs) was prepared by electrospinning technique. The precursor solution was pushed out by the syringe with the rate of 0.1 mm/min at the spinning voltage of 15 kV.

PAANMs were rolled by the electric roller press, and the thickness was reduced to approximately 50% of the original thickness to obtain physically crosslinking PAANMs (PAANMs-PC). The PAANMs and PAANMs-PC were imidized at 150 °C, 250 °C and 350 °C for 1 h, respectively, to obtain PINMs and PINMs-PC. The chemical reagents were obtained by mixing acetic anhydride, isoquinoline and DMAc with molar ratio of 1:0.5:0.1, where the acetic anhydride and the isoquinoline were acted as dehydrating agent and catalyst, respectively. Isoquinoline (97%) was provided by Shanghai Aladdin Biochemical Technology Co. (Shanghai, China). Acetic anhydride (AR) was provided Chengdu Kolon Chemical Co. (Chengdu, China) PAANMs-PCs were dipped into the above chemical reagents and kept at 60 °C, 70 °C and 80 °C for 10 min, and then imidized at the same conditions to obtain chemical crosslinking PINMs (PINMs-CC). Then, the three samples were carbonized at 700 °C for 0.5 h under argon, and activated by NH_3_ to obtain samples recorded as CNMs, CNMs-PC, and CNMs-CC, respectively.

### 2.2. Characterization

The morphologies of carbon membranes were observed by a scanning electron microscope (SEM, phenom pharos-type, Phenom-World, Eindhoven, Netherlands). Fourier-transform infrared spectroscopy was measured on a Nicolet iS50 infrared spectroscopy instrument (FT-IR, Nicolet iS50, Thermo fisher Scientific, MA, USA). Thermogravimetric analyzer (TGA, STA 2500, NETZSCH, Selb, Germany) was employed to analyze the pyrolysis behaviors of samples in flowing N_2_ at a heating rate of 10 °C/min from 30 to 800 °C. The tensile mechanical properties of samples (5 × 2 cm) were tested using an intelligent electronic tension machine (XLM, Labthink, Jinan, China) with a speed of 50 mm/min. The X-ray diffraction (XRD, D8 ADVANCE, Bruker, Karlsruhe, Germany) was performed by using Cu Kα radiation (λ = 0.154nm) with 2θ from 10 to 80 °. The Bruner-Emmet-Teller (BET) specific surface areas of the samples were obtained by the isothermal nitrogen adsorption and desorption measurements on a surface analyzer (TriStar II 3020, Micromeritics, Atlanta, USA). The electric conductivity was tested by a four-point probes resistivity tester (HPS2662, Changzhou, China). Contact angle measuring instrument (WAM-100, Shenzhen, China) was employed to identify the wettability. The variations of element state in the electrodes before and after NH_3_ treatment were investigated using X-ray photoelectron energy spectra (XPS, ESCALAB 250Xi, Thermo Fisher Scientific, MA, USA).

### 2.3. Electrochemical Measurement

The as-prepared CNMs, CNMs-PC and CNMs-CC (a diameter of 14 mm) were used as work electrodes without any binders to assemble CR2025-type coin cells with 1 M LiPF_6_/EC: DMC electrolyte in an argon-filled glovebox. Counter electrode was Li foil (15.6 × 0.45 mm). Celgard 2400 polypropylene film was used as separator. The amount of electrolyte added was 100 µL. Electrochemical performance was performed on Neware Battery Test System (Neware CT-4000, Shenzhen, China). Cyclic voltammetry (CV) tests with a rate of 0.1 mV s^−1^ between 0.01 and 1.5 V and electrochemical impedance spectroscopy (EIS) tests in the frequency range from 100 kHz to 0.1 Hz were performed on Princeton Applied Research Spectrometer (Versa STAT 3, DE, USA).

## 3. Results and Discussion

**Figure 2:**
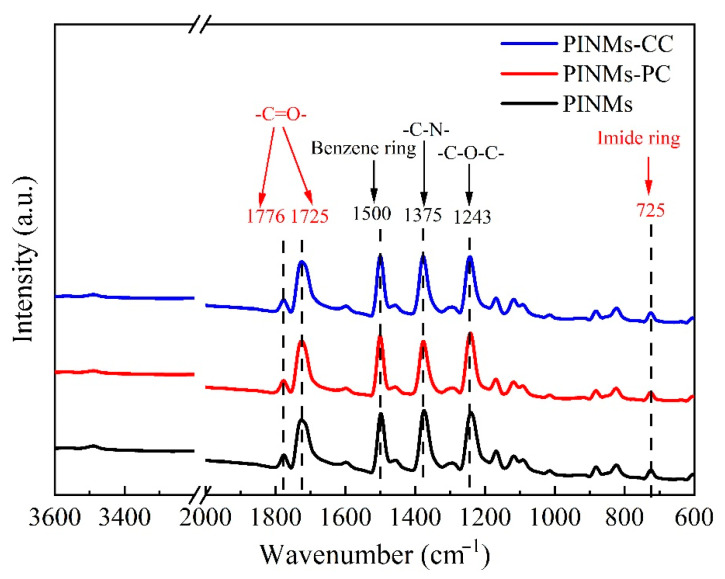
FT−IR spectrum of PINMs, PINMs−PC and PINMs−CC.

Figure 2 shows the infrared characterization spectrum of the as-prepared PI membranes. On the one hand, the appearance of the characteristic absorption peaks of the five-membered imine ring in the main chain of PI proves that PI has been successfully obtained, which is the C=O symmetric stretching vibration absorption peak on the imide ring at 1776 cm^−1^. The asymmetric stretching vibration peak of imide bond corresponds to 1725 cm^−1^. The peak at 1375 cm^−1^ belongs to C-N stretching vibration absorption peak on imide ring. The stretching vibration peak of C-O-C bond and the stretching vibration peak of the representative imide ring are the peaks of 1243 and 725 cm^−1^, respectively. It is proved that the samples are completely imidized and the structure is consistent with the target polymer. On the other hand, the peaks at 1562–1685 cm^−1^ and the peak at 3440 cm^−1^ representing the free amine groups in the system almost disappeared after imidization, including unreacted amine groups and free amine groups on the molecular chain. The disappearance of the characteristic absorption peaks of PAA indicates that the PAA membranes have finished imidization. The characteristic absorption peaks of the chemical bonds and characteristic functional groups of the polymer do not change with the crosslinking treatment conditions.

**Figure 3:**
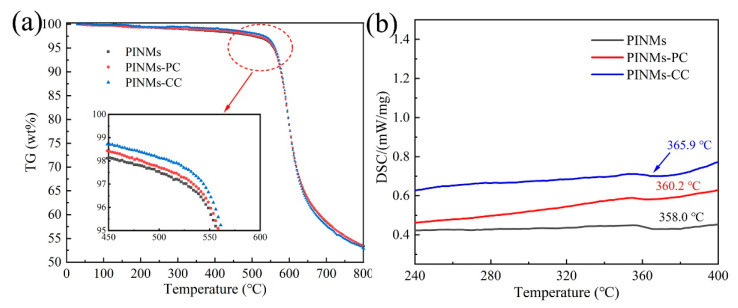
(**a**) TGA curve and (**b**) DSC curve of PI membranes at a heating rate of 10 °C/min under nitrogen atmosphere.

The effects of crosslinking treatment on the thermal stability of PI membranes were analyzed by TG and DSC tests in N_2_ atmosphere. Figure 3a is the thermal weight loss curve of samples under nitrogen protection, exhibiting the inherent thermal stability of PI membranes. Firstly, an obvious plateau exists before 500 °C, suggesting that PI has good heat resistance. Secondly, a sharp drop of the curve from 500 °C to 700 °C shows the significant loss of mass, which means that PI membranes undergo a violent decomposition reaction. Notably, at 500 °C, PINMs, PINMs-PC and PINMs-CC lose 2.47%, 2.27% and 1.83% by weight, respectively, indicating that the crosslinking structure strengthens the molecular chains and slightly improves the heat resistance performance. The formation of the crosslinking structure is beneficial to increase the glass transition temperature (Tg) of membranes as seen from DSC curve (Figure 3b). Notably, the mass retention rates of CNMs, CNMs-PC and CNMs-CC are 58.45%, 58.61% and 57.74%, respectively, at 700 °C. The thermogravimetric analysis indicates that morphology of nanofibers has changed from the initial cylindrical structure to a flat structure, which is more conducive to the evaporation of non-carbon atoms in the carbonization process.

**Figure 4:**
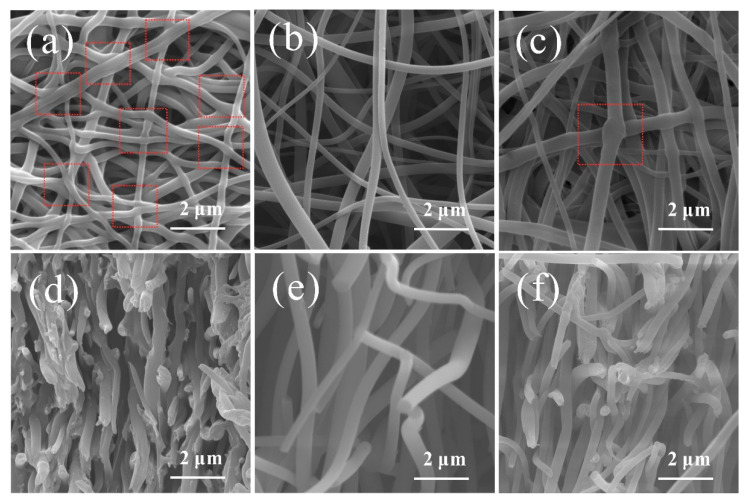
Surface and cross-section morphologies of (**a**,**d**) CNMs-CC, (**b**,**e**) CNMs, and (**c**,**f**) CNMs-PC.

The surface and cross-section morphologies of CNMs-CC, CNMs, and CNMs-PC are shown in Figure 4. Many crosslinking points are formed in the CNMs-CC sample as shown in Figure 4a due to the triple crosslinking strategy, which are marked with red dotted lines. The 3D network structure of CNMs-CC facilitates the wetting of electrolyte and electron transport. From the cross-section morphology shown in Figure 4d, a dense lamellar structure is formed between layers, which is beneficial to the improvement of mechanical strength. As references, Figure 4b,c,e,f are the surface and cross-section morphologies of CNMs and CNMs-PC, respectively. There are many smooth cylindrical fibers in CNMs as shown in Figure 4b,e. The fibers are loosely lapped and stacked together to form fluffy voids, exhibiting the morphological characteristics of non-woven fabrics. After physical rolling, the fibers of CNMs-PC display flattened forms and very few crosslinking points could be observed on the cross-section of the fibers.

**Figure 5:**
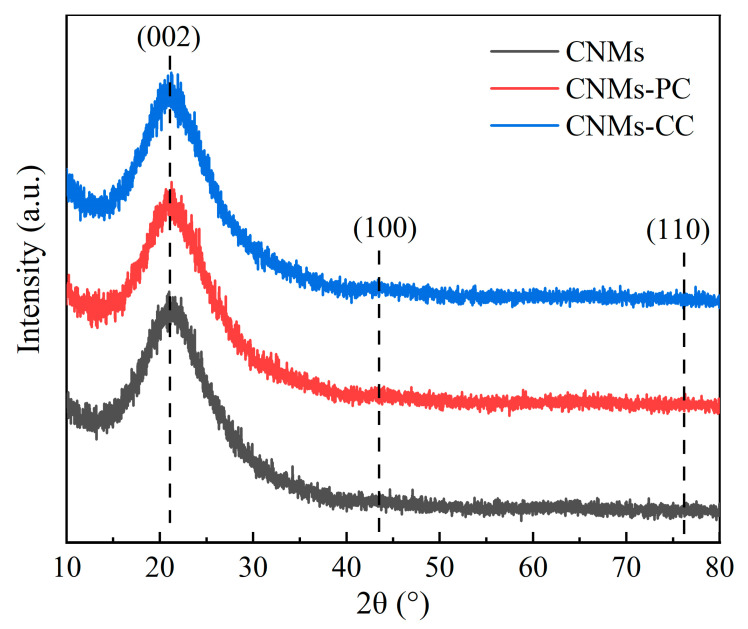
X-ray diffraction patterns of CNMs, CNMs-PC and CNMs-CC.

The crystal structures of CNMs, CNMs-PC and CNMs-CC are observed by X-ray diffraction patterns (XRD), as shown in Figure 5. The diffraction peaks of CNMs-CC are similar to those of CNMs and CNMs-PC. There is no obvious difference in the structural order of the aggregated state, indicating that the crystal structure is less affected the triple crosslinking strategy. “Steamed bun” like peaks at approximately 2θ = 20° are considered as diffuse diffraction peaks caused by amorphous structures in the molecules, suggesting that the composition of the prepared samples is still dominated by amorphous carbon [34,35,36]. Since the carbonization temperature is not high enough relative to the graphitization temperature, the samples have not yet turned into the structure of graphite. The radial arrangement of molecules is disrupted, with tiny crystals, an undeveloped structure and irregular arrangement. In addition, the polyimide of PMDA/ODA owns the planar molecular structure, and the presence of ether bonds disturbs the orientation regularity of the molecular chain. The axial lateral stacking density of the molecular chain is small, which is not conducive to obtaining graphite microcrystals with large crystallite dimensions.

**Figure 6:**
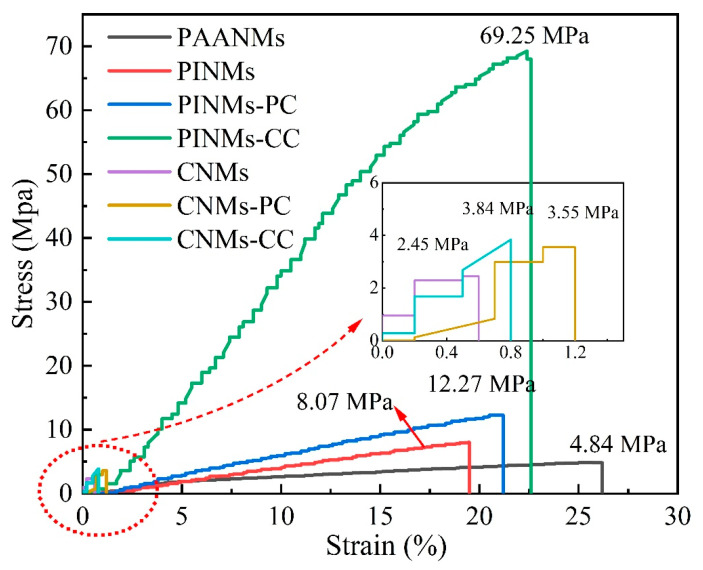
Stress–strain curves of samples.

In general, the mechanical strength can be improved by making the structure dense through physical rolling. As shown in Figure 6, the tensile strength of the original PAANMs is only 4.84 MPa. However, the tensile strengths of PINMs and PINMs-PC are 8.07 and 12.27 MPa, respectively. Furthermore, the tensile strength of PINMs-CC increases to as high as 69.25 MPa, demonstrating that the triple crosslinking approach effectively and substantially improves the mechanical properties of PINMs through the formation of a large number of physical intersection points. By previous thermogravimetric analysis, the PI precursor shows excellent heat resistance due to the retention of the aromatic amide structure, which leads to the fiber membranes maintaining high mechanical properties. After carbonization, there is a dramatic decrease in mass and gas release that occurs inside the membranes due to the breakage and reorganization of molecular bonds, resulting in a significant decrease in mechanical properties, which is consistent with the results in Figure 3 and Figure 5. Nevertheless, the mechanical strength of the CNMs-CC with the value of 3.84 MPa is still highest, which proves that the novel triple crosslinking strategy on carbon nanofiber membranes is effective and efficient.

**Figure 7:**
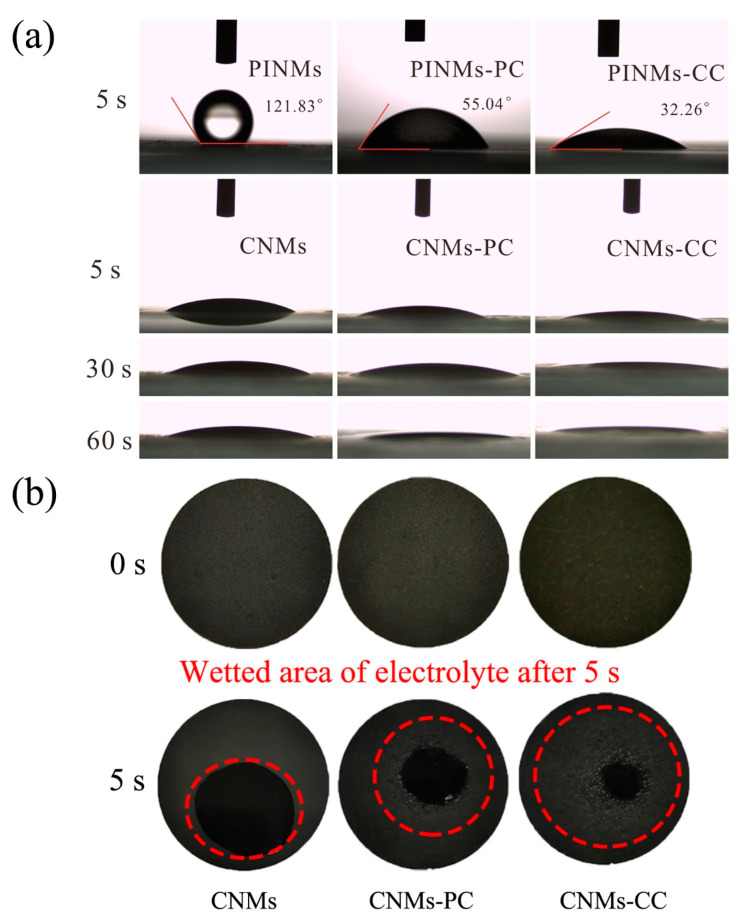
(**a**) Contact angle determination of PINMs, PINMs-PC, PINMs-CC, CNMs, CNMs-PC and CNMs-CC and (**b**) photographs of electrolyte wettability for CNMs, CNMs-PC and CNMs-CC.

In general, excellent compatibility between the liquid electrolyte and the electrode will reduce the internal resistance and facilitate ion conduction, which is expected to further improve the electrochemical performance of the LIBs. Therefore, the contact angle test was carried out to characterize the wettability of as-prepared samples, in which the deionized water as the dropping liquid, as shown in Figure 7a. It is obvious to see that the contact angles of the PINMs, PINMs-PC and PINMs-CC after 5 s are 121.83°, 55.04° and 32.26°, respectively, as shown in Figure 7a. After carbonization, the wettability of the corresponding carbon membranes can be further improved due to their higher surface energy. CNMs-CC with a triple crosslinking structure exhibits the best wettability. To further investigate the wettability of samples to electrolyte in battery applications, liquid electrolytes were employed to drop on the surface of the CNMs, CNMs-PC and CNMs-CC. The wetting areas of the droplets on the surfaces of samples were observed to characterize the differences in wettability, as shown in Figure 7b. It may be related to the effect of decomposition on the membrane surface and the size of the micro-crystals formed during carbonization. However, the specific reasons need to be further studied.

**Figure 8:**
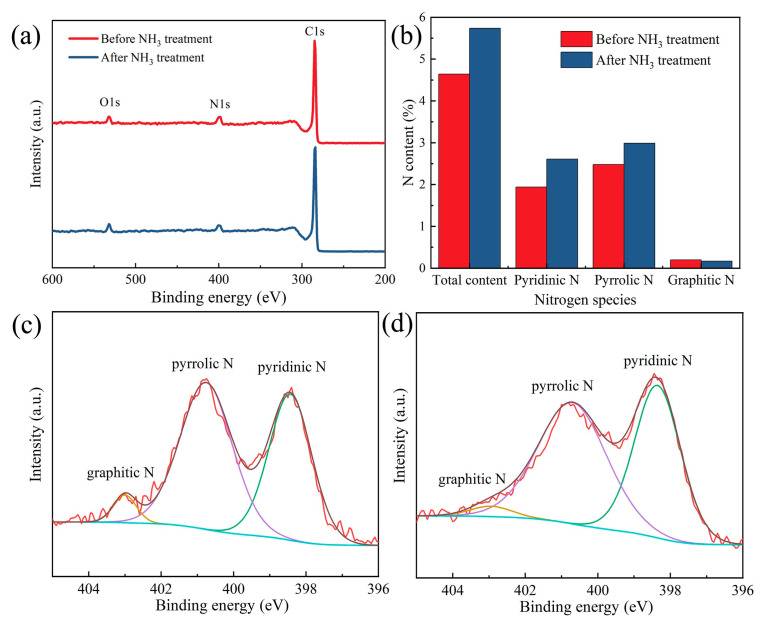
(**a**) XPS spectra, (**b**) ratios of N content, N1s spectra for (**c**) CNMs-CC before NH_3_ treatment and (**d**) CNMs-CC after NH_3_ treatment.

As shown in Figure 8, XPS is employed to describe the elemental species and the valence of N. Figure 8a shows the survey scan spectra of C, O and N. As expected, the N content is increased by the NH_3_ treatment (Figure 8b). The N content of the CNMs-CC with NH_3_ activation (5.74%) is higher than that of CNMs-CC before NH_3_ treatment (4.64%). The configurations of N before and after NH_3_ treatment are depicted in Figure 8c,d, respectively. It can be classified into three configurations by split-peak fitting, including graphitic nitrogen, pyrrole nitrogen and pyridine nitrogen at 398.4, 400.8 and 403.1 eV, respectively. Pyridine N and graphitic N can increase the electronic conductivity due to sp^2^ hybridized orbitals [37,38,39]. Therefore, the surface treatment with NH_3_ can increase the surface activity of carbon fibers while etching the surface, indicating that NH_3_ is of great benefit for the improvement of electrochemical properties [40,41,42].

As discussed before, PI molecular chain contains many benzene rings and aromatic heterocyclic structures, and the molecular chain breaks to form benzene ring radicals during heat treatment. Further increasing the carbonization temperature, the benzene ring radicals condense and polymerize with each other to form a hexagonal carbon network plane. The electron orbitals overlap a lot, which makes the PI-based carbon fiber have certain electrical conductivity [43,44]. The conductivities of CNMs, CNMs-PC and CNMs-CC were listed in Table 1 with the values of 10.22, 22.30, and 41.91 S/m, respectively. Obviously, the CNMs-CC shows excellent electron conductivity, which is owing to the unique triple crosslinking structure and the significant increase in crosslinking points.

**Figure 9:**
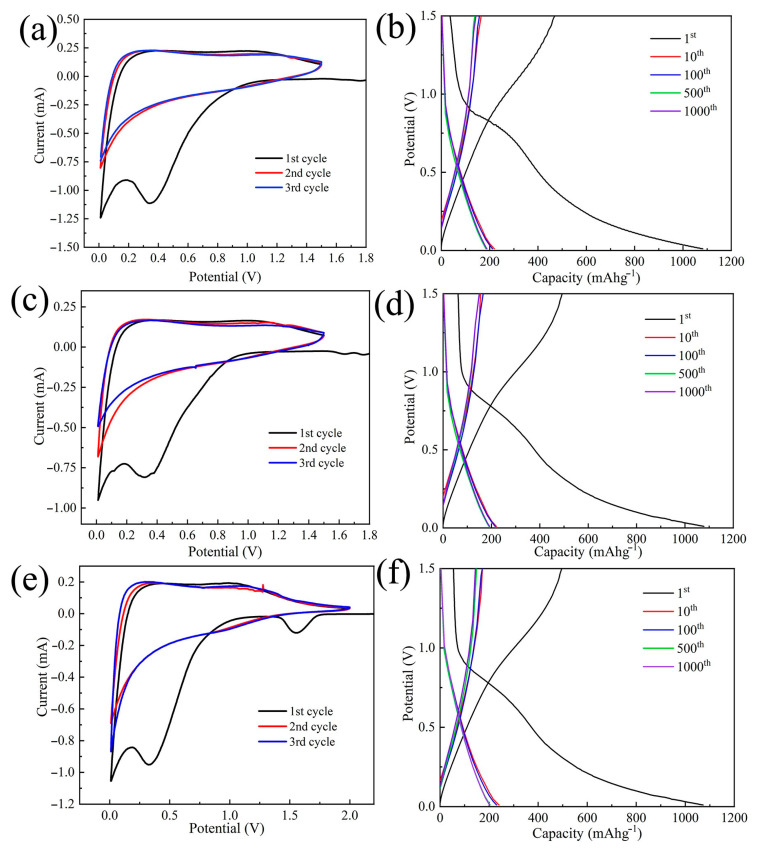
CV curves of (**a**) CNMs, (**c**) CNMs−PC (**e**) CNMs−CC at 0.1 mVs^−1^ and discharge/charge profiles for (**b**) CNMs, (**d**) CNMs−PC and (**f**) CNMs−CC at a current density of 1A g^−1^.

As shown in Figure 9, CNMs-CC, CNMs and CNMs-PC display typical characteristics of hard carbon materials by CV and charge/discharge tests. Figure 9a,c,e show the CV curves of CNMs, CNMs-PC and CNMs-CC, of which these samples were used as anodes and metallic Li served as counter electrode. Two irreversible peaks of CV curve for CNMs-CC appeared at 0.4 and 1.5 V during the first cycle, which may be associated with the formation of solid electrolyte interphase (SEI), as the peaks disappeared during the last two cycles. After the first cycle, the curves are almost overlapped, exhibiting stable lithium-ion insertion-extraction behavior. Likewise, the charge/discharge curves of CNMs, CNMs-PC and CNMs-CC are shown in Figure 9b,d,f. In line with the CV curve, there is a voltage drop plateau at 0.2–0.8 V in the first cycle due to the SEI film [45,46,47]. Similarly, compared with CNMs and CNMs-PC, voltage plateau regions of the charge–discharge profiles for CNMs-CC also disappeared in the subsequent cycles. The capacity degradation happens dominantly during the initial cycle. The detailed parameters of the carbon electrodes during charging and discharging are summarized in Table 2.

The initial discharge capacity of CNMs-CC (1072.70 mAh g^−1^) is amazing, which is almost three times higher than that of commercial graphite anodes. However, owing to the highly irreversible formation of SEI film, the initial Coulomb efficiencies of CNMs, CNMs-PC and CNMs-CC are only 49.40%, 49.42% and 50.06%, respectively. Therefore, the tests results reveal that the novel triple crosslinking strategy on carbon nanofiber membranes as flexible electrodes is beneficial to improve the first Coulomb efficiency to some extent.

**Figure 10:**
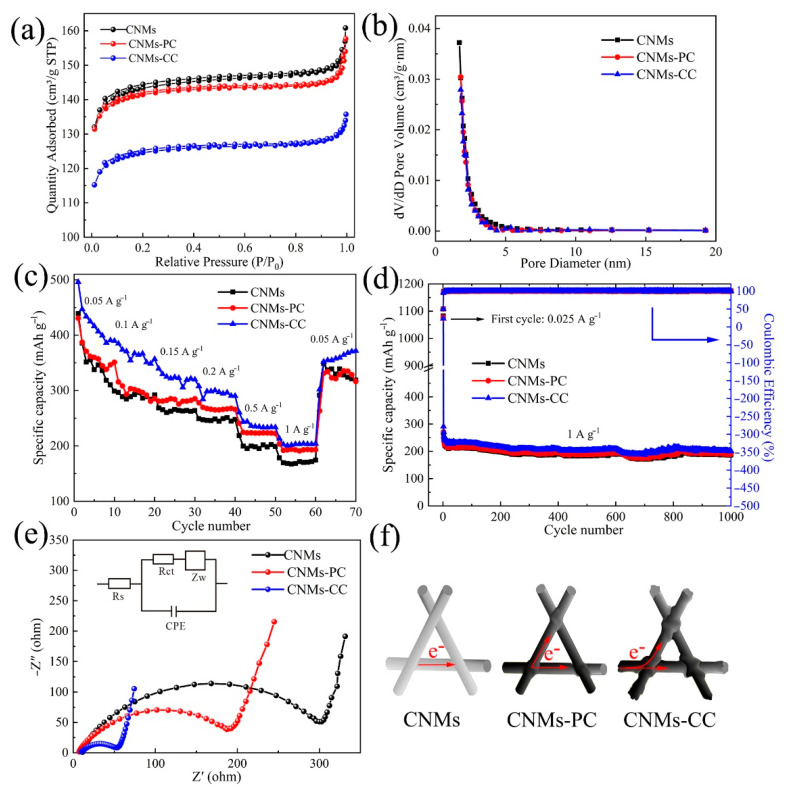
(**a**) N_2_ adsorption−desorption isotherms, (**b**) BJH adsorption pore size distribution, (**c**) rate performance, (**d**) cycling performance, (**e**) EIS (Nyquist plots), measured at an amplitude of 5 mV over a frequency range of 100 kHz−0.1 Hz, and (**f**) illustration of electron transfer for CNMs, CNMs−PC and CNMs−CC.

To characterize the porous texture, among the most important physicochemical properties of carbon-based materials, the N_2_ adsorption-desorption isotherms and pore size distribution curves are depicted in Figure 10a,b. All three curves are typed I adsorption curves. The adsorption and desorption plateaus decrease sequentially, which indicates that CNMs-CC has the lowest specific surface area. By calculation, the BET surface areas of CNMs, CNMs-PC and CNMs-CC are 459.35, 452.84, and 398.43 m^2^/g and the average pore sizes are 0.25, 0.24, and 0.20 cm^3^/g, respectively. Meanwhile, the sample surface exists mainly as a microporous structure, which can be judged by the almost horizontal curves with the *P*/*P*_0_ value between 0.2 and 0.8. Thus, the high specific surface area of electrode offers larger electrolyte/electrode interfaces and more active sites for Li^+^ adsorption during charging and discharging. Meanwhile, SEI film is associated with the specific surface area of the carbon membranes, so the generated irreversible capacity loss strongly depends on the specific surface area [48].

The rate performances of CNMs, CNMs-PC and CNMs-CC at various current densities are plotted in Figure 10c. CNMs-CC exhibits high stability with capacities of 495, 384, 339, 307, 260 and 213 mAh g^−1^ at current densities of 0.05, 0.1, 0.15, 0.2, 0.5, and 1 A g^−1^, respectively. However, the reversible capacity of CNMs and CNMs-PC are poorer than that of CNMs-CC at all current densities. Notably, CNMs-CC shows excellent cycling performance after 1000 cycles (Figure 10d), even at a high current density. The three electrodes exhibit excellent cycling stability and all show similar capacity decay rates. CNMs, CNMs-PC and CNMs-CC deliver discharge capacities of 265.51, 271.08 and 290.87 mAh g^−1^, respectively. After 1000 cycles, they remain 188.68, 191.86, and 201.38 mAh g^−1^, respectively. Compared to CNMs and CNMs-PC, CNMs-CC shows best rate and cycling performances, which can be attributed to the synergistic effects of its stable triple crosslinking structure, good wettability, electrical conductivity, and smaller impedance. The crosslinking network structure provides 3D fast electron paths, as evidenced by four-point probe measurements (Table 2).

EIS measurements are performed as shown in Figure 10e. The curve consists of a semicircle related to the charge transfer impedance and a diagonal line corresponding to the resistance to Li^+^ diffusion [49,50]. By comparison, the semicircle of CNMs-CC is smallest, demonstrating higher electron conductivity, which is particularly critical to enhance electrode rate performance. The specific values were obtained by fitting with ZVIEW, as shown in Table 3. Moreover, the triple crosslinking network structure of CNMs-CC is more compact, which can form 3D electron transport pathway through the intersection points caused by the novel triple crosslinking strategy, as demonstrated in Figure 10f. Furthermore, the Li^+^ transport distance is shortened to improve the electrochemical performance of LIBs. Compared with the conventional electrodes, the self-supporting flexible carbon nanofiber membranes does not require grinding and coating processes, greatly reducing the process flow. When used as electrodes, the flexible carbon nanofiber membranes not only serve as lithium-ion charge and discharge of the active material carrier, but also serve as fluid collector for electron collection.

**Figure 11:**
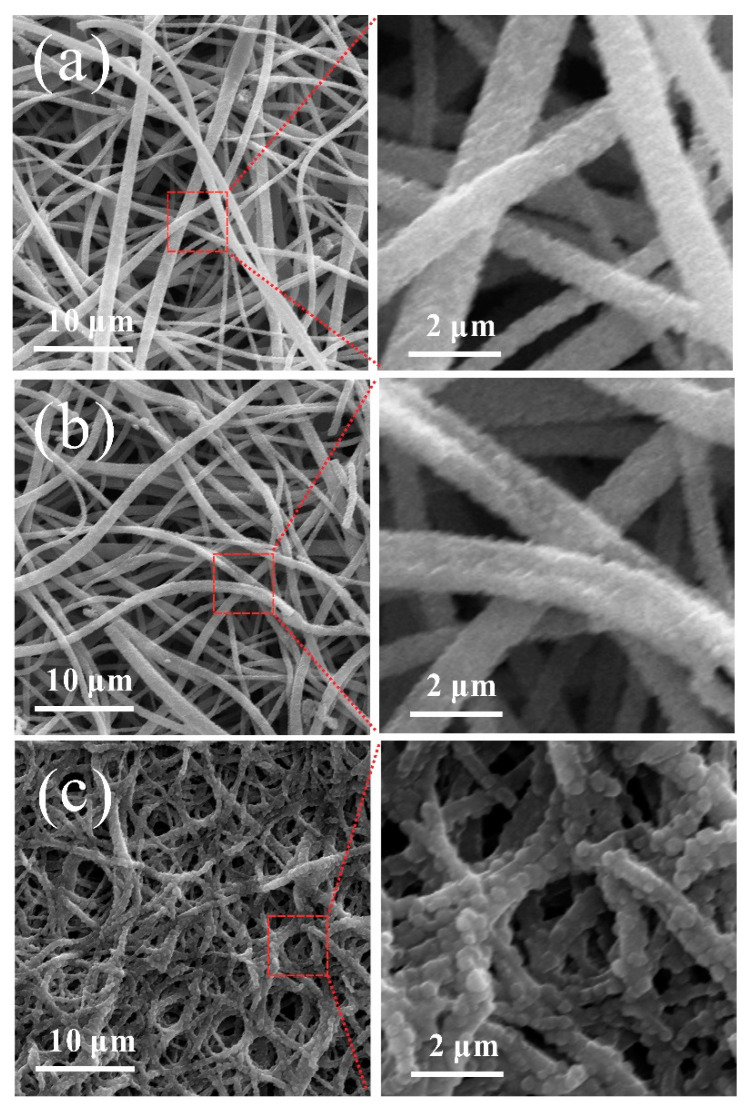
SEM images of (**a**) CNMs, (**b**) CNMs-PC and (**c**) CNMs-CC after 1000 cycles.

To further explore the changes in CNMs-CC morphology after charging and discharging, Figure 11 shows the SEM morphologies of CNMs, CNMs-PC, CNMs-CC as flexible electrodes after 1000 cycles. Compared with CNMs and CNMs-PC, the surface of CNMs-CC is more tightly covered by small particles and the SEI film is more uniform. The fibers of CNMs-PC remain flat and the morphologies of CNMs are very consistent, basically maintaining the cylindrical fiber shape before cycling. The surface is covered by small particles, which is related to SEI film. As is known to all, a stable SEI film is crucial to the battery performance, because it can effectively prevent the damage of electrodes by co-embedding of solvent molecules and improve the cycling performance. As seen in Figure 11, the freestanding 3D network structure significantly suppresses the volume expansion effect during lithium embedding and de-embedding. Therefore, the CNMs-CC with stable SEI film exhibits more excellent electrochemical performance, which has been proved in the characterization of rate and cycle performances.

## 4. Conclusions

In summary, a triple crosslinking strategy was put forward to fabricate carbon nanofiber membranes via synergistic action of pre-rolling, solvent and chemical imidization. The novel crosslinking structure grants CNMs-CC many advantages, including excellent mechanical properties and wettability, especially electrical conductivity and fast charge transfer capability. The CNMs-CC exhibits excellent electrochemical performances, such as a good reversible capacity of 495 mAh g^−1^, superior rate capability with current densities from 0.05 to 1 A g^−1^ and an ultra-long cycling stability of 201.38 mAh g^−1^ over 1000 cycles at 1 A g^−1^. Furthermore, it can withstand the repeated impacts of lithium ions during charging and discharging owing to strong structural stability. Therefore, considering their excellent performance, flexibility, simple synthesis process and low cost, CNMs-CC could be promising binder-free anodes for flexible batteries and supercapacitors.

## Figures and Tables

**Table 1:** The Resistivity and electrical conductivity of samples.

Samples	Square Resistivity (Ω)	Resistivity (Ω·cm)	Electric Conductivity (S/m)
CNMs	1087.36	9.78	10.22
CNMs-PC	738.27	4.48	22.30
CNMs-CC	588.52	2.39	41.91

**Table 2:** The parameters of charge/discharge cycle data of samples.

Samples	Initial Discharge Specific Capacity/(mAh g^−1^)	Initial Charge Specific Capacity/(mAh g^−1^)	Initial Coulomb Efficiency	10th/(mAh g^−1^)	100th (mAh g^−1^)	1000th (mAh g^−1^)
CNMs	1082.32	534.64	49.40%	219.61	211.66	188.68
CNMs-PC	1078.14	532.86	49.42%	220.44	215.31	191.86
CNMs-CC	1072.70	536.95	50.06%	240.38	230.17	201.38

**Table 3:** The parameters by fitting EIS spectra of samples.

Samples	Rs (Ω)	Rct (Ω)
CNMs	10.9	296.3
CNMs-PC	5.8	198.6
CNMs-CC	9.3	48.1

## Data Availability

The data that support the findings of this study are available from the corresponding author upon reasonable request.
